# Mechanical Properties and Water Stability of High Ductility Magnesium Phosphate Cement-Based Composites (HDMC)

**DOI:** 10.3390/ma14123169

**Published:** 2021-06-09

**Authors:** Hu Feng, Yang Wang, Aofei Guo, Xiangyu Zhao

**Affiliations:** School of Civil Engineering, Zhengzhou University, Zhengzhou 450001, China; fenghu@zzu.edu.cn (H.F.); wangyeccs@126.com (Y.W.); zhaoxiangyu1208@126.com (X.Z.)

**Keywords:** magnesium phosphate cement, ambient curing age, water immersion age, water/binder ratio, magnesium oxide/potassium dihydrogen phosphate ratio, mechanical property, water stability

## Abstract

In this study, the compressive test and four-point flexural test were carried out to explore the water stability as well as mechanical properties of high ductility magnesium phosphate cement-based composites (HDMC). The effects of ambient curing age (7 d and 28 d), water immersion age (7 d, 28 d, and 56 d), water/binder ratio (W/B), and magnesium oxide/potassium dihydrogen phosphate ratio (M/P) on the mechanical properties (compressive strength, first-crack strength, ultimate flexural strength, ductility index, and toughness index) and water stability of the HDMC were examined. The results showed that the 28-day ambient curing could lead to higher retention rates of strength, ductility, and toughness than 7-day ambient curing, indicating better water stability; however, it did not result in significant improvement in the mechanical properties of the HDMC. As the water immersion age increased, the mechanical properties of the HDMC with 7-day ambient curing showed an obvious downward trend; the mechanical properties of the HDMC with 28-day ambient curing did not show an obvious decrease and even could be increased in many cases, especially when the water immersion age was 56 days; and the change of water stability was consistent with that of the mechanical properties. If all indexes and their corresponding retention rates were considered comprehensively, the W/B ratio of 0.16 and the M/P ratio of 5 seemed to be the optimum values for the HDMC. The scanning electron microscopy analysis confirmed that the water immersion had a large adverse effect on the HDMC and thus reduced their mechanical properties.

## 1. Introduction

Magnesium phosphate cement (MPC) refers to an inorganic cementitious material that is produced through the acid-base neutralization reaction of MgO and phosphate [1,2,3]. In previous studies, dead-burned MgO and ammonium phosphate were used as raw materials to produce magnesium ammonium phosphate cement, but the release of ammonia during the hydration process hindered its wide application. Then researchers used potassium dihydrogen phosphate to replace ammonium phosphate to produce MPC. The MPC has excellent characteristics such as fast setting and hardening, high early strength [4], high bonding strength with old concrete [5], small drying shrinkage [5], solidification of liquid wastes containing heavy metals [6], excellent wear resistance [7], frost resistance performance [8], high-temperature resistance [9,10], etc. It has a promising engineering application prospect for highways, bridges, airport runways, and other places with rapid repair needs. It also has research prospects for 3D printing [11], alternative biomaterials for hard tissue applications [12], fiber-reinforced inorganic polymer composites [13], etc.

Similar to ordinary Portland cement-based materials, the MPC-based composites have the characteristics of brittleness and low strain capacity. The addition of fibers can increase the strength and toughness of the MPC-based composites and reduce the number and width of matrix cracks due to their bridging effect. It was reported that the addition of fibers (e.g., steel fibers, glass fibers, basalt fibers, and polypropylene fibers) can improve the compressive strength, flexural strength, ductility, and toughness of MPC-based composites and make them exhibit strain-hardening behavior [14,15,16,17,18,19,20,21,22,23]. In addition, polyvinyl alcohol fibers (PVA), as a kind of hydrophilic fibers, can effectively enhance the tensile ductility and flexural properties of cement-based composites due to their high tensile strength, high elastic modulus, and good acid and alkali corrosion resistance [24,25,26,27]. Lee et al. found that PVA fibers could inhibit the shrinkage and cracking of composite materials [28]. Wang et al. believed that PVA fibers could enhance the displacement extension and fracture energy [29]. Therefore, PVA fibers are used in this study to prepare high ductility MPC-based composites (HDMC).

The mechanical properties of the HDMC can be impaired when immersed in water for a long time because the MPC can be degraded despite its excellent mechanical properties [30,31,32]. The main factors affecting the water stability of MPC are the ambient curing age, magnesium oxide/potassium dihydrogen phosphate (M/P) ratio, water/binder (W/B) ratio, etc. For the curing method, prolonging air pre-curing time can make the MPC hydration reaction more complete and increase its initial strength before being immersed in water, resulting in a positive effect on water stability [33]. Wang found that the strength loss rate of specimens with 28 days of air curing was significantly lower than that of specimens with 7 days of air curing while being immersed in water [34]. For the M/P ratio, Li et al. studied the MPC prepared with different M/P ratios and found that when the M/P ratio increased from 2:1 to 8:1, the compressive strength loss of the specimens after immersion in water for 28 d decreased from 65.3% to 12%, which indicated that a higher M/P ratio could lead the specimens to have better water stability [31]. However, if there is too much MgO, the hydration reaction rate is so fast that it is difficult to form dense reaction products, which may decrease the matrix strength [35,36]. For the W/B ratio, it can change the solidification time, phase composition, and compressive strength of MPC-based composites, and normally a higher water content leads to more pores in the matrix, affecting the water stability negatively [37,38]. Therefore, in this study, the factors of ambient curing age, M/P ratio, and W/B ratio are taken into consideration.

The high ductility magnesium phosphate cement-based composite (HDMC) has superior ductility and strength and can be used in many practical engineering projects, whereas its water stability has not been fully understood. In this study, the HDMC was prepared by combining the MPC with PVA fibers (1.6% volume fraction). Given that fly ash can improve the water stability of the MPC [39], it was incorporated to replace 20% cement by weight. This study mainly consists of two parts. First, based on a reference mix proportion, the effects of ambient curing age (duration in the ambient curing room) and water immersion age (duration in water after ambient curing) on the water stability, together with mechanical properties, of the HDMC were studied. Then, to optimize the mix proportions, the effects of the M/P ratio and the W/B ratio on the water stability, together with mechanical properties, of the HDMC were examined. Lastly, the scanning electron microscopy (SEM) test was conducted to provide some in-depth analysis.

## 2. Materials and Methods

### 2.1. Raw Materials

Magnesium phosphate cement (MPC) was prepared from a mixture of dead-burned magnesium oxide (MgO, marked as M), potassium dihydrogen phosphate (KH_2_PO_4_, marked as P), and borax (Na_2_B_4_O_7_∙10H_2_O, marked as B). The dead-burned magnesium oxide (MgO) powder with a specific surface area of 315.7 m^2^/kg was used in the study. Its chemical composition is shown in Table 1.

Two kinds of industrial-grade potassium dihydrogen phosphates (P) in the form of white crystalline powder were mixed with a mass ratio of 1:1 and then were used as an acidic compound. The particle size ranges of these two potassium dihydrogen phosphates were 180–315 μm and 425–600 μm, which were denoted as fine P and coarse P, respectively. The borax (B) was used as a cement retarder to delay the setting time. The physical properties of the M, the P, and the B are summarized in Table 2.

The quartz sand with a particle size range of 120–180 μm was used as fine aggregates, with its technical parameters shown in Table 3.

The first-grade fly ash (FA) was selected, with its chemical composition shown in Table 4.

**Figure 1:**
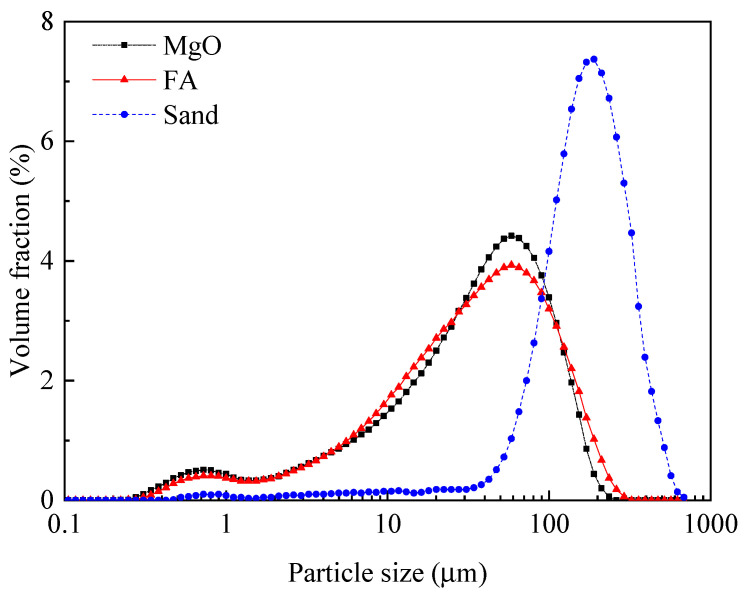
Particle size distribution of MgO, FA, and quartz sand.

**Figure 2:**
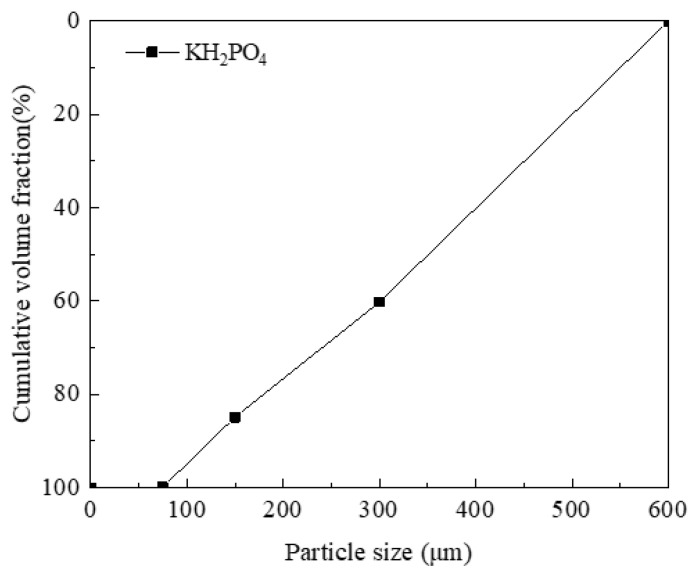
Particle size distribution of KH_2_PO_4_.

The PVA fibers used were produced by Kuraray Company of Japan, with their specific performance indicators shown in Table 5. The particle size distribution of MgO, quartz sand, and FA is shown in Figure 1. The particle size distribution of KH_2_PO_4_ is shown in Figure 2.

### 2.2. Mix Proportion

Based on the experimental study in the authors’ research group, when the mass ratio of sand to binder (MPC + FA), denoted as S/B, is 0.2 and the borax dosage is 6% by mass of MgO, the mechanical properties and ductility of specimens are relatively good. Therefore, in this study, for all specimens, these two numbers are selected. The PVA fiber volume ratio is 1.6%, and the FA is used to replace 20% (by mass) MPC. A reference mix proportion is adopted, in which the mass ratio of water to binder (MPC + FA), denoted as W/B, is 0.16, and the molar ratio of MgO to KH_2_PO_4_, denoted as M/P, is 4. To explore the effects of ambient curing age (duration in the ambient curing room) and water immersion age (duration in water following the ambient curing) on the HDMC, two ambient curing ages (7 days and 28 days) and three water immersion ages (7 days, 28 days, and 56 days) are considered on the reference mix proportion (W/B = 0.16 and M/P = 4). To explore the effects of W/B and M/P on the HDMC, four W/B ratios (0.14, 0.16, 0.18, and 0.20) and four M/P ratios (3, 4, 5, and 6) are considered while both the ambient curing age and the water immersion age are 7 days. The mix proportions are shown in Table 6.

### 2.3. Specimen Preparation

Firstly, the weighed magnesium oxide, potassium dihydrogen phosphate, quartz sand, borax, and fly ash were added to the single-horizontal shaft concrete mixer and mixed for 120 s to make the mixture uniform. Secondly, water was added and mixed for 120 s, during which PVA fibers were added several times to ensure their uniform dispersion. Thirdly, the mixture continued to be mixed for another 120 s after the addition of PVA fibers. Lastly, the mixture was cast into 50 × 50 × 50 mm^3^ plastic molds and 400 × 100 × 15 mm^3^ steel molds to prepare cubic specimens for compressive test and thin plate specimens for four-point flexural test, respectively.

The cast specimens were stored in the curing room (temperature: 25 ± 2 °C, relative humidity: 45 ± 5%) for 2h, after which the molds were removed. After that, the specimens continued to be stored in the curing room to a certain age (7 days or 28 days) and then were immersed in a water tank for a certain age (7 days, 28 days, or 56 days).

### 2.4. Test Methods

#### 2.4.1. Compressive Test

**Figure 3:**
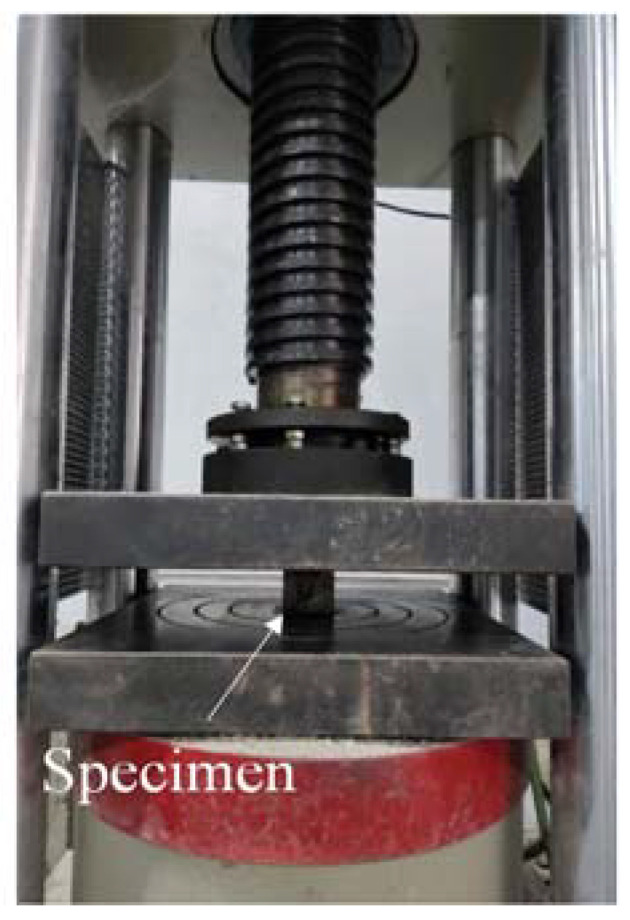
Diagram of compressive test.

The compressive test was carried out on the cubic specimens (50 × 50 × 50 mm^3^) according to ASTM C109 [44]. Three samples were tested for each group, and the average value was adopted. The test instrument is a pressure testing machine (200T), as shown in Figure 3, with a loading speed of 0.9 kN/s.

#### 2.4.2. Four-Point Flexural Test

**Figure 4:**
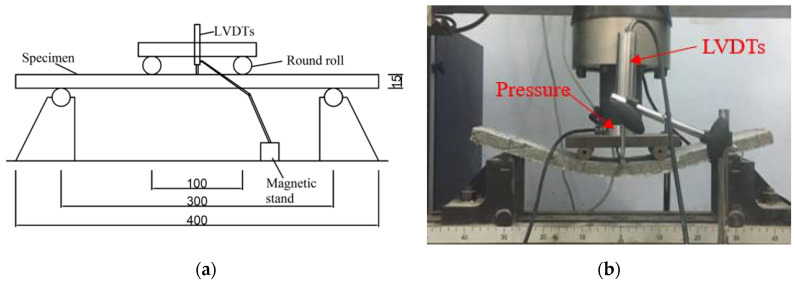
(**a**) Schematic diagram and (**b**) loading diagram of four-point flexural test.

The four-point flexural test was conducted on the thin plate specimens, as shown in Figure 4. The specimen size was selected based on the study from Xu et al. [45]. The loading speed was adjusted to 0.3 mm/min for continuous loading. The collected mid-span deflection values and the corresponding load values of the specimens were plotted into a load-deflection curve.

#### 2.4.3. Scanning Electron Microscopy (SEM) Test

The scanning electron microscopy (SEM) test was conducted to examine the samples at the microscopic level. The samples were broken and cut into 1 mm^2^ size using a precision gas cutting machine and then put into the field emitted scanning electron microscope (Zeiss, Oberkochen, Germany) for the SEM test. The effects of the water immersion on the matrix and its bonding with fibers were analyzed by observing the changes in the microstructure of the samples.

### 2.5. Flexural Performance Evaluation

**Figure 5:**
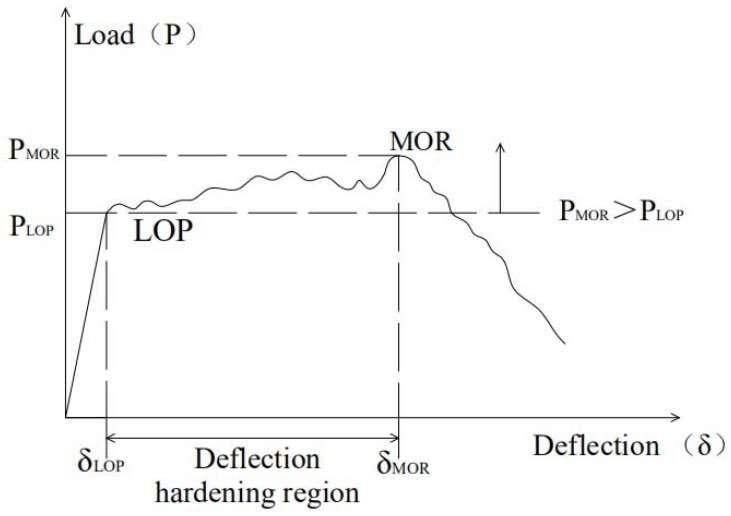
Typical strain hardening curve of fiber-reinforced cement-based composites.

Figure 5 shows a typical strain hardening curve of a fiber-reinforced cement-based composite material. The first cracking point is the position where the load-deflection curve changes from linear to nonlinear, which is called the limit of proportion (LOP) according to ASTM C1018 [46]. The load and deflection at the LOP are called cracking load (*P_LOP_*) and cracking deflection (*δ_LOP_*), respectively. The modulus of rupture (MOR) is defined as the point at which the load-deflection curve begins to soften. The load and deflection at the MOR are called ultimate load (*P_MOR_*) and ultimate deflection (*δ_MOR_*), respectively.

(1) Strength index

The first-crack strength, *f_LOP_* (MPa), and ultimate flexural strength, *f_MOR_* (MPa), are calculated using Equations (1) and (2), respectively.

(1) $$f_{LOP}=P_{LOP}\frac{L}{bh^{2}}$$

(2) $$f_{MOR}=P_{MOR}\frac{L}{bh^{2}}$$

where *P_LOP_* is the cracking load (N), *P_MOR_* is the ultimate load (N), *L* is the span length of the thin plate specimens (mm), and *b* and *h* are the width and height of the thin plate specimens (mm), respectively.

(2) Ductility index

The ductility index (*DI*) is defined as the difference between the ultimate deflection (*δ_MOR_*) and the cracking deflection (*δ_LOP_*), as shown in Equation (3). The greater the difference is, the better the ductility of specimens can be.

(3) $$DI=\delta_{MOR}-\delta_{LOP}$$

where *δ_MOR_* is the ultimate deflection (mm), and *δ_LOP_* is the cracking deflection (mm).

(3) Toughness index

The toughness index (*T_peak_*) is defined as the difference between the area under the load-deflection curve up to the ultimate deflection and the area up to the cracking deflection, as shown in Equation (4). The larger the difference is, the better the toughness of specimens can be.

(4) $$T_{peak}=S_{MOR}-S_{LOP}$$

where *S_MOR_* is the area under the load-deflection curve up to the ultimate deflection (kN·mm), and *S_LOP_* is the area under the load-deflection curve up to the cracking deflection (kN·mm).

### 2.6. Water Stability Evaluation

Water stability was evaluated by defining the strength retention rate, ductility retention rate, and toughness retention rate. The larger the value is, the better the water stability of specimens is.

The strength retention rate (*k_c_*) is calculated using Equation (5).

(5) $$k_{c}=f_{c2}/f_{c1}\times 100\%$$

where *f_c_*_1_ and *f_c_*_2_ are the strength (including compressive strength, first-crack strength, and ultimate flexural strength) (MPa) of the specimens without water immersion and with water immersion, respectively.

The ductility retention rate (*k_DI_*) is calculated using Equation (6).

(6) $$k_{DI}=\frac{DI_{2}}{DI_{1}}\times 100\%$$

where *DI*_1_ and *DI*_2_ are the ductility indexes of the thin plate specimens without water immersion and with water immersion, respectively.

The toughness retention rate (*k_T_*) is calculated using Equation (7).

(7) $$k_{T}=\frac{T_{2}}{T_{1}}\times 100\%$$

where *T*_1_ and *T*_2_ are the toughness indexes of the specimens without water immersion and with water immersion, respectively.

## 3. Results and Discussion

### 3.1. Effect of Ambient Curing Age and Water Immersion Age

#### 3.1.1. Compressive Strength and Its Retention Rate

**Figure 6:**
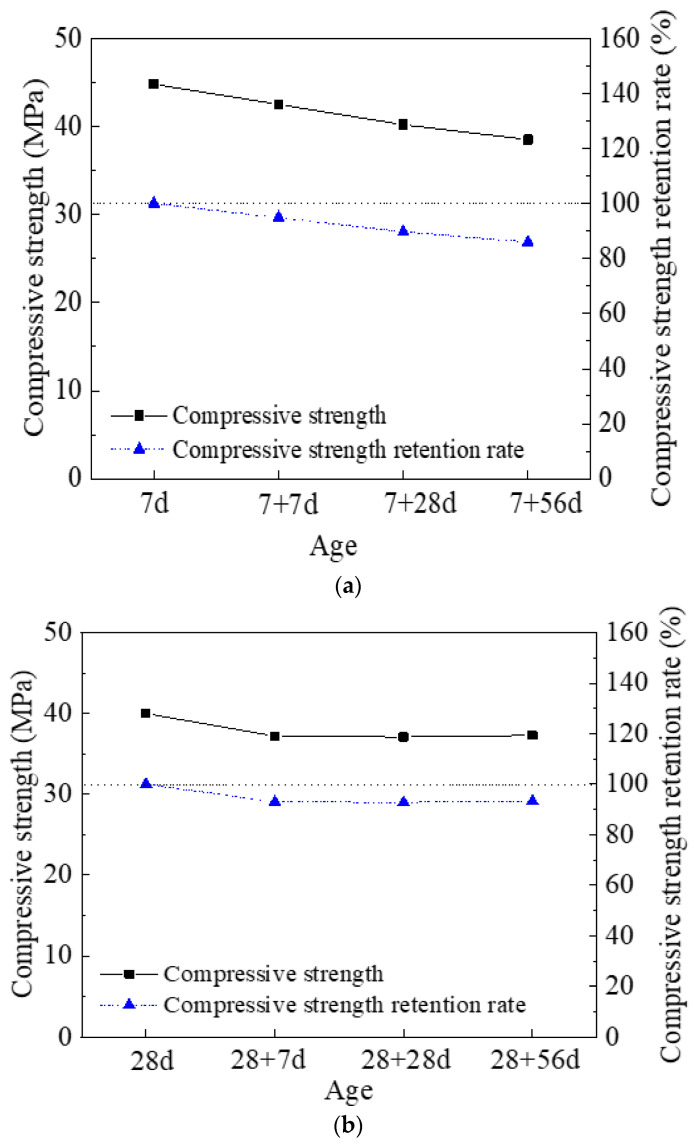
Compressive strength and its retention rate of HDMC with (**a**) 7 days and (**b**) 28 days of ambient curing (Note: the standard deviation is within 10% of the average value, which is also applicable to Figure 7, Figure 8, Figure 9, Figure 10 and Figure 11).

Figure 6a,b shows the compressive strength and compressive strength retention rate of the HDMC with 7-day and 28-day ambient curing, respectively. For non-immersed HDMC, the 7-day compressive strength is 44.8MPa, which is slightly higher than the 28-day compressive strength (40MPa). Also, for immersed HDMC, regardless of water immersion age, the 7-day ambient curing can result in higher compressive strength than the 28-day ambient curing. Figure 6 also shows that as the water immersion age increases from 0 to 56 days, the compressive strength of the HDMC with 7-day ambient curing shows an obvious downward trend; however, the compressive strength of the HDMC with 28-day ambient curing decreases to a small extent, especially when the water immersion age changes from 7 to 56 days.

Figure 6 shows that the compressive strength retention rate of the HDMC with 28-day ambient curing is slightly higher than that of the HDMC with 7-day ambient curing, which indicates that the increase of ambient curing age can enhance the water stability of HDMC. This is because increasing the ambient curing age can make the hydration of MPC more complete, the structure more compact, the number of phosphates not involved in the reaction reduced, and the dissolution of phosphates after water immersion decreased significantly. In addition, Figure 6a shows that for the HDMC with 7-day ambient curing, the compressive strength retention rate shows a downward trend as the water immersion age increases, reaching 86% when the water immersion age is 56 days. Figure 6b shows that for the HDMC with 28-day ambient curing, the compressive strength retention rate does not change too much as the water immersion age increases from 7 to 56 days, remaining at about 93%. This indicates that the hydration of the HDMC with 28-day ambient curing is more completed, so the water immersion age has little effect on the compressive strength.

#### 3.1.2. Strength Index, Ductility Index, and Toughness Index and Corresponding Retention Rates

**Figure 7:**
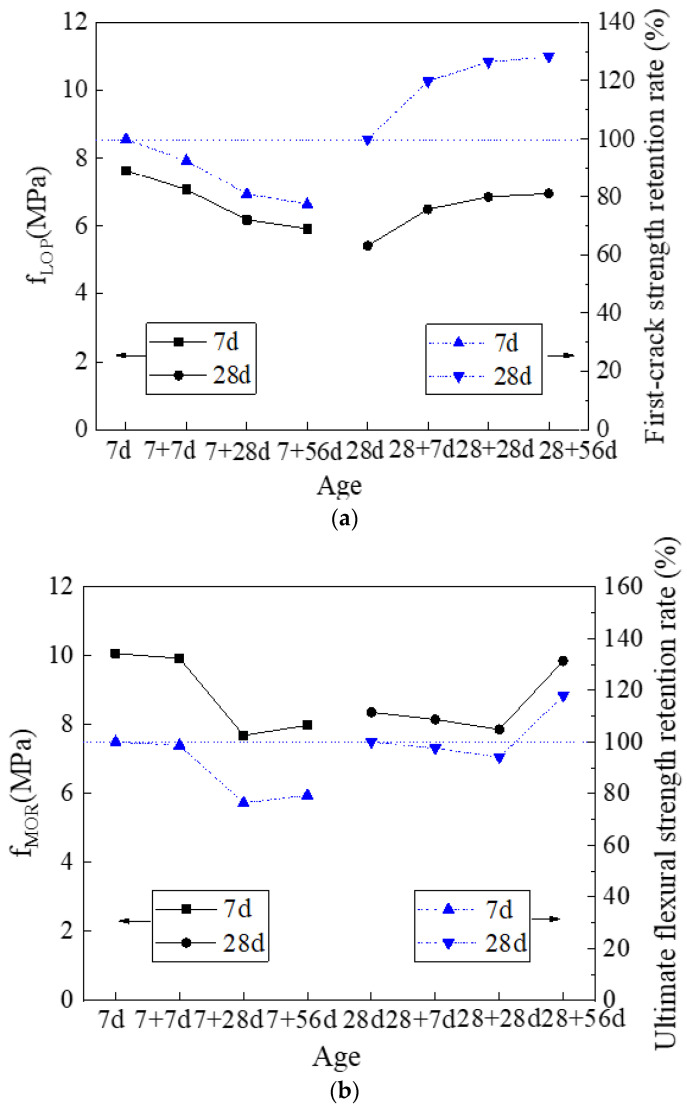

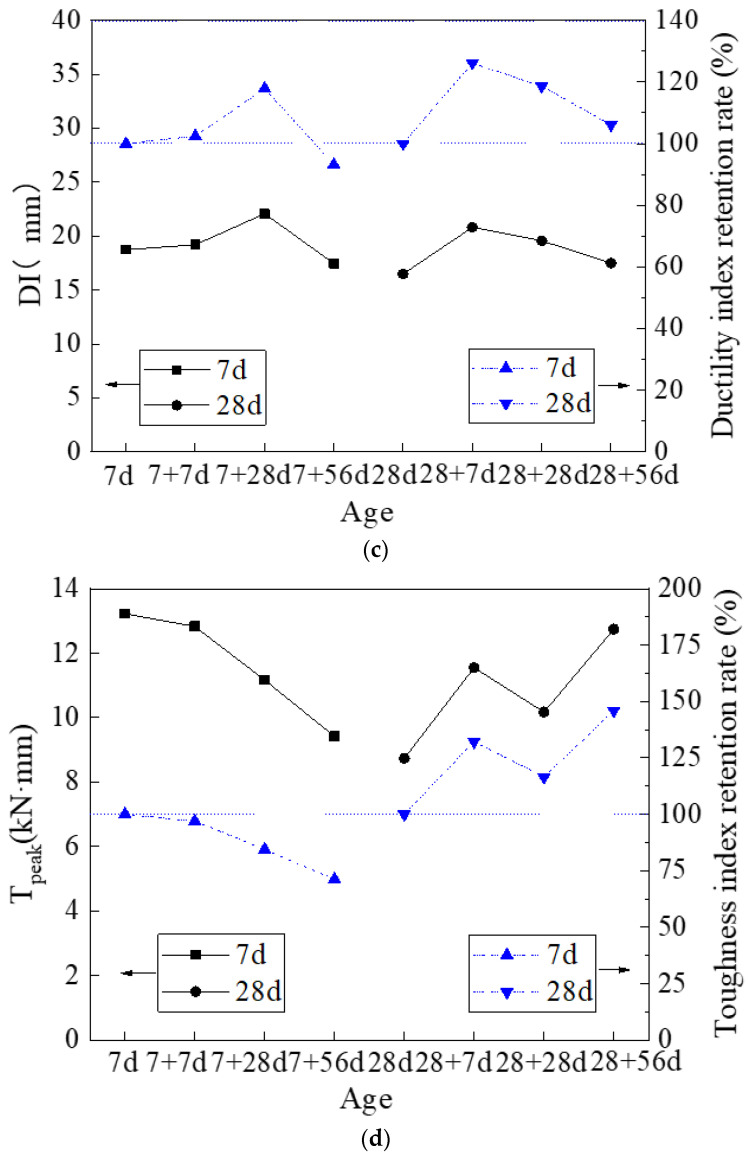
(**a**) First-crack strength, (**b**) ultimate flexural strength, (**c**) ductility index, and (**d**) toughness index, together with their retention rates, of HDMC with different ambient curing ages and water immersion ages.

The strength index (first-crack strength and ultimate flexural strength), ductility index, and toughness index of the HDMC with different ambient curing ages and water immersion ages can be calculated by using Equations (1)–(7). The effect of ambient curing age and water immersion age on these parameters is shown in Figure 7. It is shown that compared to the HDMC with 7-day ambient curing, the HDMC with 28-day ambient curing have higher first-crack strength and ultimate flexural strength only when they are immersed in water for 28 days or 56 days, higher ductility index only when they are immersed in water for 7 days or 56 days, and higher toughness index only when they are immersed in water for 56 days. In general, the 28-day ambient curing does not lead to significantly improved strength index, ductility index, and toughness index compared to 7-day ambient curing.

Figure 7 also shows that for the HDMC with 7-day ambient curing, as the water immersion age increases, the first-crack strength, ultimate flexural strength, and toughness index gradually decrease except for that the 56-day water immersion age leads to a slightly higher ultimate flexural strength than 28-day water immersion age; the ductility index increases first and then decrease, reaching the highest value of 22.07 mm when the water immersion age is 28 days. For the HDMC with 28-day ambient curing, as the water immersion age increases, the first-crack strength shows a gradual increase; the ultimate flexural strength decreases first and then increases, reaching the highest value of 9.85 MPa when the water immersion age is 56 days; the ductility index first increases and then decreases, reaching the highest value of 20.81 mm when the water immersion age is 7 days; and the toughness index shows an ascending trend on the whole, reaching the maximum value of 12.75 kN·mm when the water immersion age is 56 days. Therefore, generally, as the water immersion age increases, the strength index, ductility index, and toughness index of the HDMC with 7-day ambient curing become worse; however, those of the HDMC with 28-day ambient curing are not so bad and even can be improved in many cases, especially when the water immersion age is 56 days.

Figure 7 shows that the retention rates of first-crack strength, ultimate flexural strength, ductility, and toughness of the HDMC with 28-day ambient curing are all higher than those of the HDMC with 7-day ambient curing, indicating better water stability. It is because, with the increase of ambient curing age, the number of unreacted phosphates in the HDMC matrix is gradually reduced; the number of hydration products is gradually increased; and the structure of the matrix is denser.

Figure 7 also shows that for the HDMC with 7-day ambient curing, with the increase of water immersion age, the retention rates of first-crack strength, ultimate flexural strength, and toughness decrease; and the retention rate of ductility increases first and then decreases, reaching the highest value of 117.84% when the water immersion age is 28 days. For the HDMC with 28-day ambient curing, with the increase of water immersion age, the first-crack strength retention rate shows a gradual increase, reaching the highest value of 128.23% after 56 days of water immersion; the ultimate flexural strength retention rate slightly decreases first and then increases, reaching the highest value of 117.87% after 56 days of water immersion; the ductility retention rate first increases and then decreases, reaching the highest value of 126.17% after 7 days of water immersion; and the toughness retention rate shows an ascending trend generally, reaching the highest value of 145.95% after 56 days of water immersion. It can be seen that the changes of strength retention rate, ductility retention rate, and toughness retention rate are consistent with those of strength index, ductility index, and toughness index. Therefore, generally, as the water immersion age increases, the water stability of the HDMC with 7-day ambient curing becomes worse; however, the water stability of the HDMC with 28-day ambient curing can be improved in many cases, especially when the water immersion age is 56 days.

### 3.2. Effect of Water/Binder (W/B) Ratio

#### 3.2.1. Compressive Strength and Its Retention Rate

**Figure 8:**
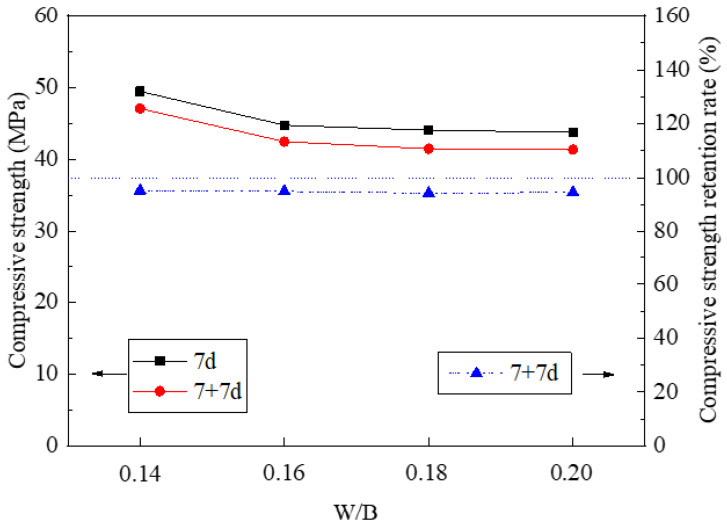
Compressive strength and its retention rate of HDMC with different W/B ratios.

Figure 8 shows the compressive strength and its retention rate of non-immersed (denoted as “7d”) and immersed HDMC (denoted as “7 + 7d”) with different W/B ratios. It can be seen that with the increase of the W/B ratio, the compressive strength of the non-immersed HDMC decreases from 49.5 to 43.8 MPa, and the compressive strength of the immersed HDMC decreases from 47.1 to 41.4 MPa. This is because when the W/B ratio is increased, the pores in the matrix can be increased due to the evaporation of excess water, thereby reducing the compressive strength of the HDMC [36,47].

From Figure 8, it can be seen that the compressive strength retention rate of HDMC is less than 1. This is because, under the water immersion condition, some hydration products are dissolved, and some phosphates dissolve to reduce the hydration degree of MgO grains and thus the formation of hydration products. Figure 8 also shows that the compressive strength retention rate of HDMC decreases slightly with the increase of the W/B ratio. When the W/B ratio is large, although the formation of hydration products decreases and some of the hydration products dissolve, increasing the matrix defects, the content of unreacted MgO grains increased, and the superimposition effect of the center particles is stronger than that of the unreacted HDMC matrix, which may make the compressive strength retention rate change little [48].

#### 3.2.2. Strength Index, Ductility Index, and Toughness Index and Corresponding Retention Rates

**Figure 9:**
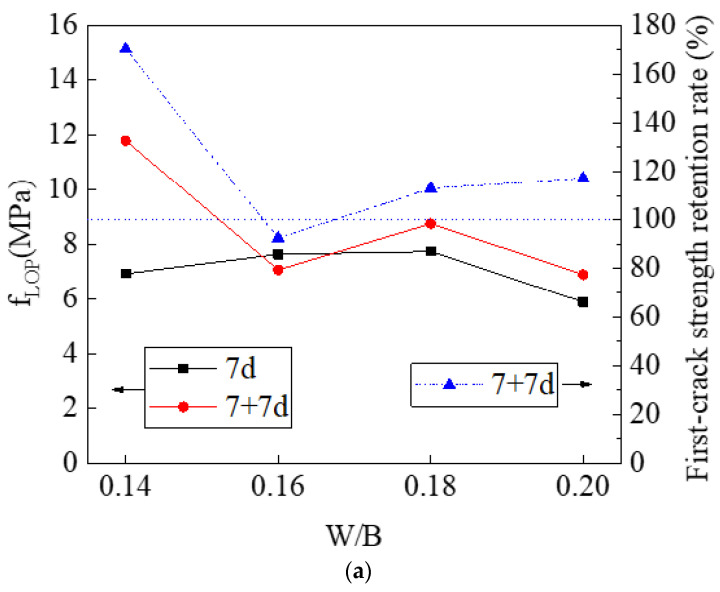

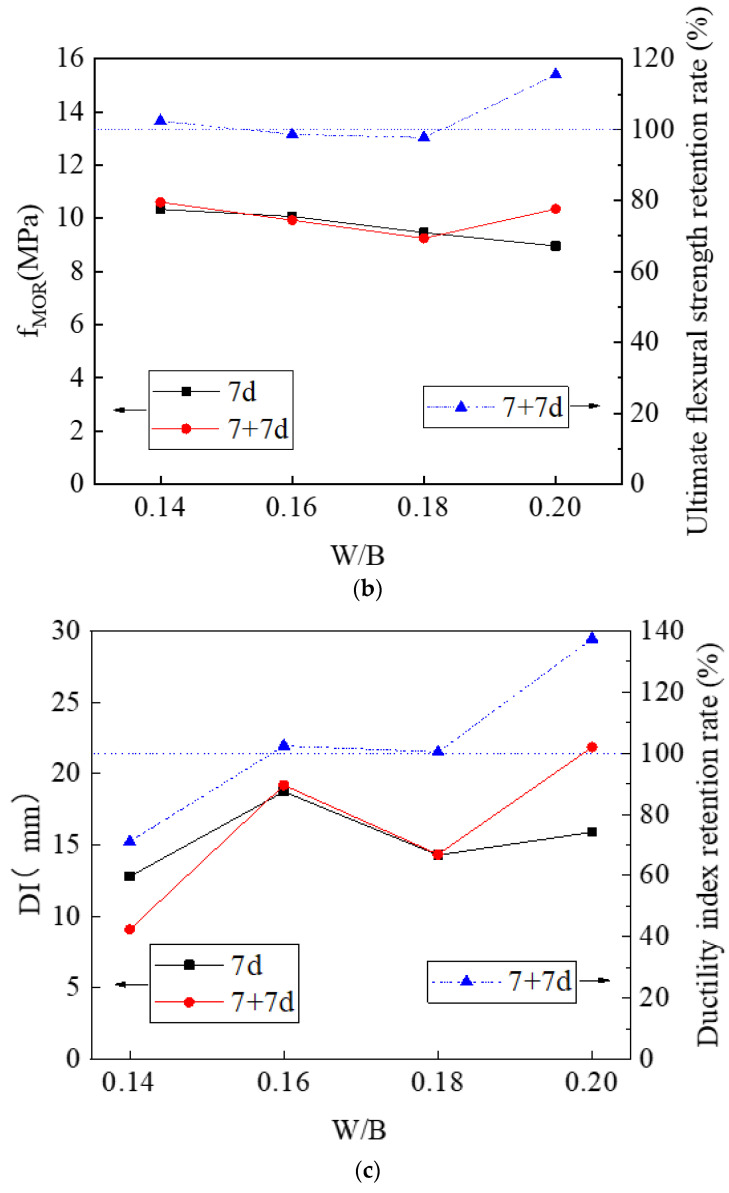

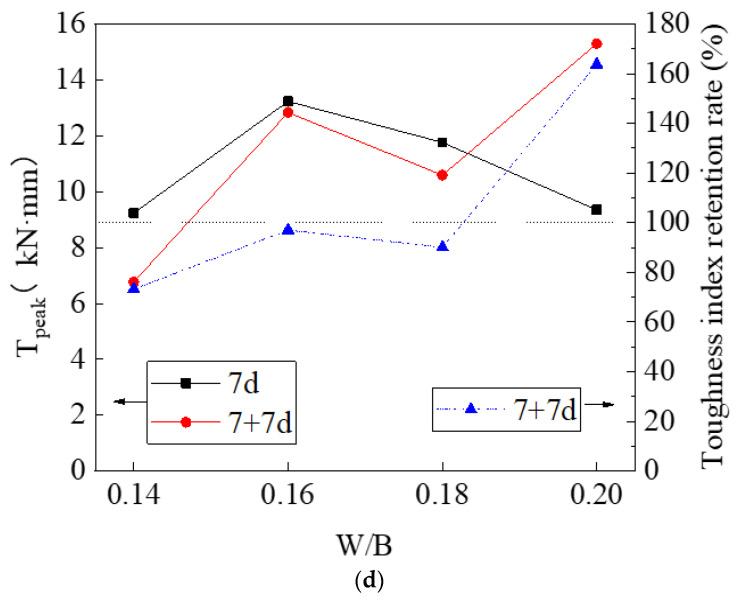
(**a**) First-crack strength, (**b**) ultimate flexural strength, (**c**) ductility index, and (**d**) toughness index, together with their retention rates, of HDMC with different W/B ratios.

The strength index (first-crack strength and ultimate flexural strength), ductility index, and toughness index, together with their retention rates, can be calculated using Equations (1)–(7). The effect of the W/B ratio on these parameters is shown in Figure 9. It can be seen that for the non-immersed HDMC, with the increase of W/B ratio from 0.14 to 0.20, the first-crack strength increases slightly first and then decreases, reaching the maximum value of 7.74 MPa when the W/B ratio is 0.18; the ultimate flexural strength decreases gradually; the ductility index first increases and then decreases, reaching the highest value of 18.73 mm when the W/B ratio is 0.16; and the toughness index first increases and then decreases, reaching the maximum value of 13.23 kN·mm when the W/B ratio is 0.16. For the immersed HDMC, with the increase of W/B ratio from 0.14 to 0.20 the first-crack strength and ultimate flexural strength show a decreasing trend; however, the ductility index and toughness index show an upward trend on the whole except when the W/B ratio is 0.18. Therefore, for non-immersed HDMC, when the W/B ratio is 0.16, the strength index, ductility index, and toughness index all can be satisfactory. For immersed HDMC, the strength index is the best when the W/B ratio is 0.14, and the ductility index and toughness index are the best when the W/B ratio is 0.20; these three indexes are moderate when the W/B ratio is 0.16.

Figure 9a,b shows that the retention rates of first-crack strength and ultimate flexural strength first decrease and then increase with the increase of the W/B ratio. When the W/B ratio is 0.14, the retention rate of first-crack strength is the highest, and when the W/B ratio is 0.20, the retention rate of ultimate flexural strength reaches the maximum value of 115.53%. Figure 9c,d shows that the retention rates of ductility and toughness show an overall upward trend as the W/B ratio increases, reaching the maximum values of 137.48% and 163.7%, respectively, when the W/B ratio is 0.20.

Generally, the strength index, ductility index, and toughness index are satisfactory when the W/B ratio is 0.16; the retention rates of strength, ductility, toughness seem to be the highest when the W/B ratio is 0.20, indicating the best water stability, followed by the W/B ratio of 0.16. Therefore, the W/B ratio of 0.16 can be the best choice for the HDMC considering both the mechanical properties and the water stability.

### 3.3. Effect of M/P Ratio

#### 3.3.1. Compressive Strength and Its Retention Rate

**Figure 10:**
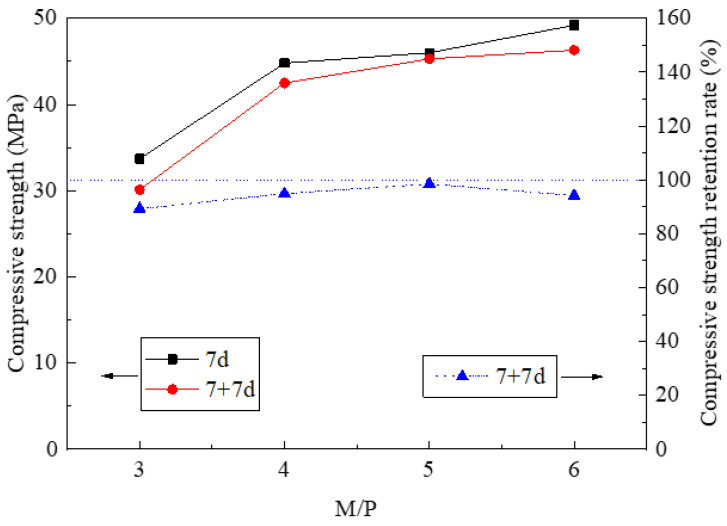
Compressive strength and its retention rate of HDMC with different M/P ratios.

Figure 10 shows the compressive strength and its retention rate of non-immersed and immersed HDMC with different M/P ratios. It can be seen that, regardless of water immersion, the compressive strength of the HDMC increases with the increase of the M/P ratio. As the M/P ratio increases from 3 to 6, the compressive strength of the non-immersed HDMC increases from 33.7 to 49.2 MPa, and the compressive strength of the immersed HDMC increases from 30.1 to 46.3 MPa.

By comparison, the compressive strength of the immersed HDMC is lower than that of the non-immersed HDMC. Therefore, the compressive strength retention rate is less than 100%, as shown in Figure 10. Besides, Figure 10 shows that the compressive strength retention rate of HDMC first increases and then decreases as the M/P ratio increases. When the M/P ratio is 5, the compressive strength retention rate reaches the maximum value of 98.5%, indicating the best water stability. This may be because a large amount of MgO makes the microstructure of the HDMC matrix relatively compact [4]. However, when the M/P ratio is 6, the compressive strength retention rate decreases slightly, which may be because that the excessive MgO content makes the early hydration reaction more fully complete, and the growth of compressive strength is more obvious after 7 days of ambient curing.

#### 3.3.2. Strength Index, Ductility Index, and Toughness Index and Corresponding Retention Rates

**Figure 11:**
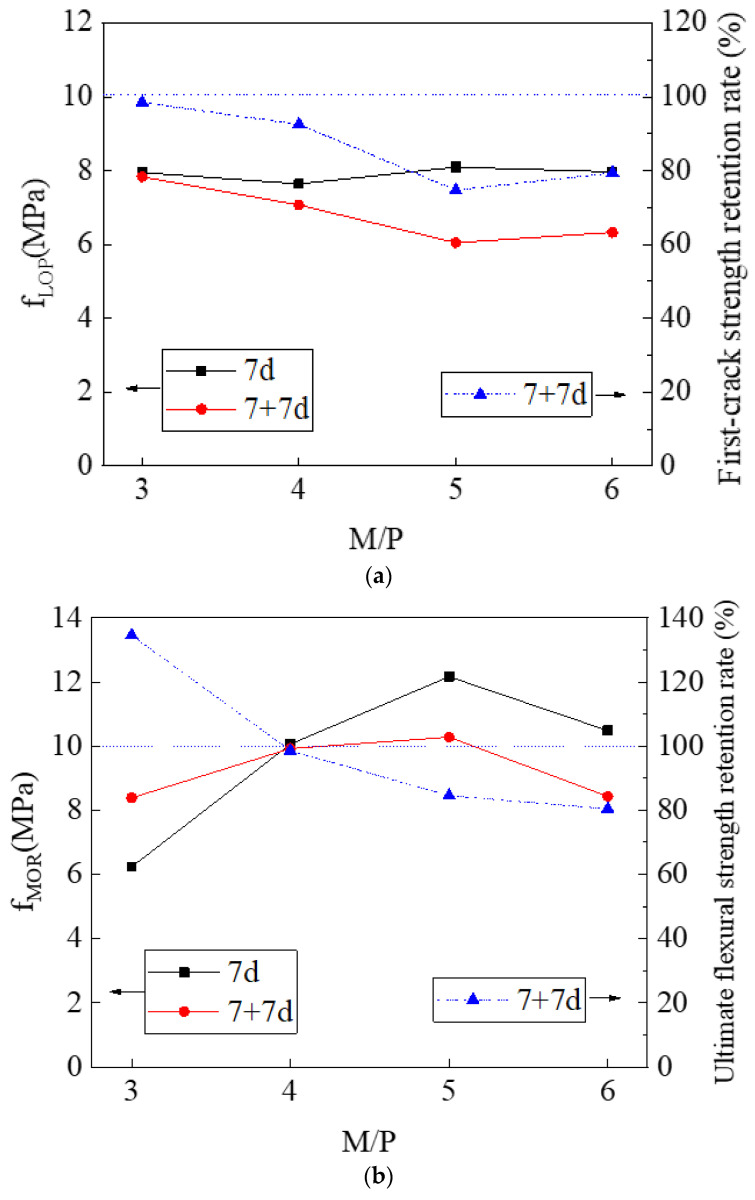

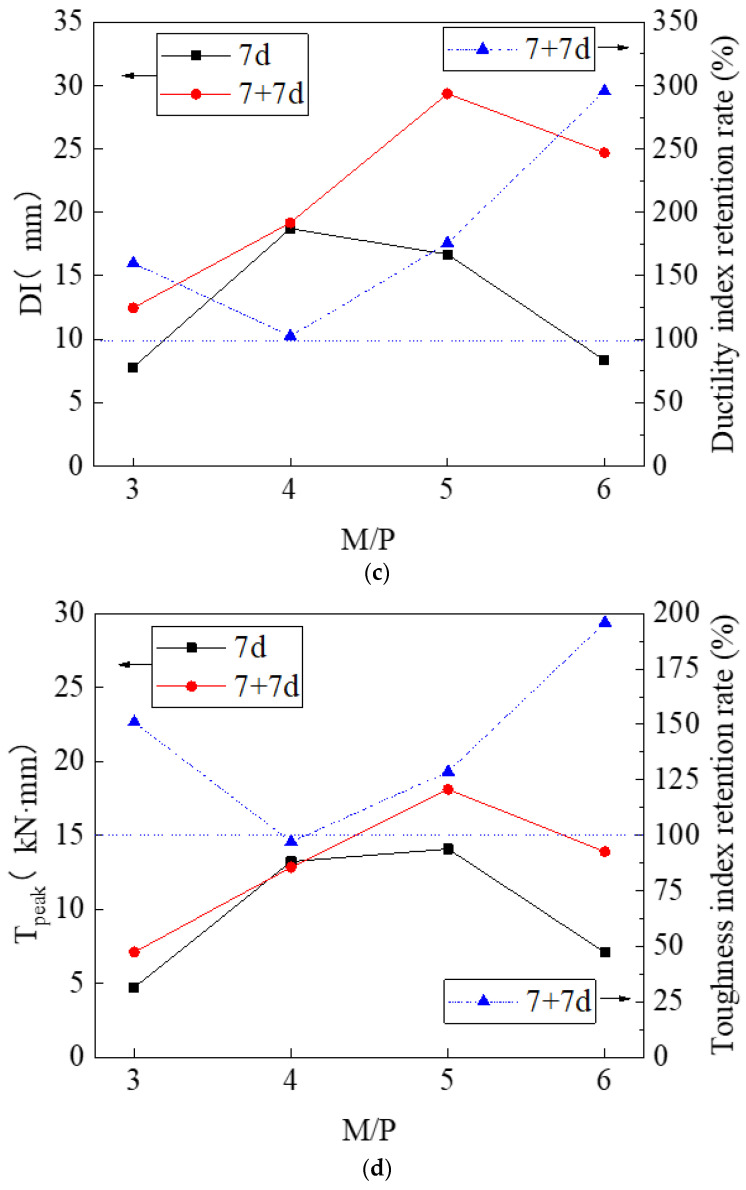
(**a**) First-crack strength, (**b**) ultimate flexural strength, (**c**) ductility index, and (**d**) toughness index, together with their retention rates, of HDMC with different M/P ratios.

Figure 11 shows the strength index (first-crack strength and ultimate flexural strength), ductility index, and toughness index of non-immersed and immersed HDMC with different M/P ratios. For the non-immersed HDMC, with the increase of M/P ratio from 3 to 6, there is no obvious change in the first-crack strength; the ultimate flexural strength first increases and then decreases, reaching the maximum value of 12.16 MPa when the M/P ratio is 5; and the ductility index and toughness index also increase first and then decrease, reaching the maximum values of 18.73 mm and 14.07 kN·mm when the M/P ratio is 4 and 5, respectively. For the immersed HDMC, as the M/P ratio increases from 3 to 6, the first-crack strength decreases gradually; the ultimate flexural strength, ductility index, and toughness index first increase and then decrease, reaching the maximum values of 10.28 MPa, 29.36 mm, and 18.11 kN·mm, respectively, when the M/P ratio is 5. It can be seen that, for both non-immersed and immersed HDMC, the strength index, ductility index, and toughness index are satisfactory when the M/P ratio is 5.

With the increase of M/P ratio from 3 to 6, the first-crack strength retention rate and ultimate flexural strength retention rate of HDMC show a decreasing trend generally, reaching the maximum values of 98.51% and 134.58%, respectively, when the M/P ratio is 3; the ductility retention rate and toughness retention rate first decrease and then increase, reaching the maximum values of 295.6% and 195.91%, respectively, when the M/P ratio is 6.

In general, when the M/P ratio is 5, the strength index, ductility index, and toughness index are satisfactory. However, the retention rate analysis indicates that the HDMC with the M/P ratio of 5 shows moderate water stability, and the best water stability is reached when the M/P ratio is 3.

### 3.4. Microstructure of HDMC

**Figure 12:**
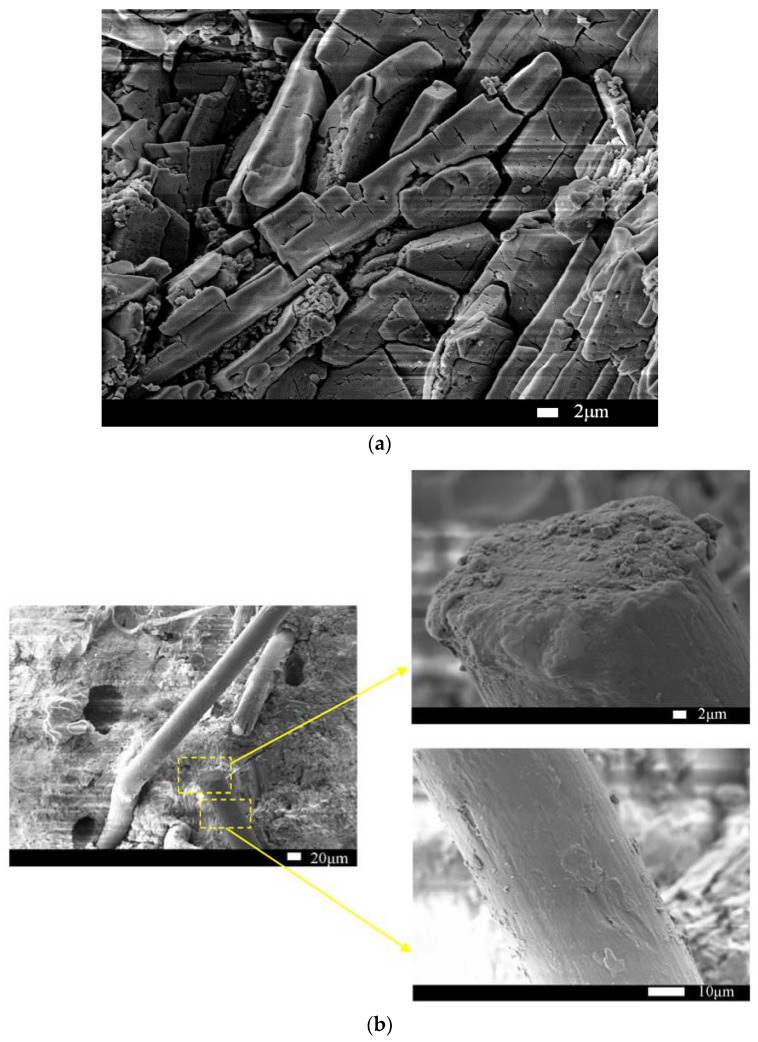
(**a**) The matrix and (**b**) its bonding with fibers for non-immersed HDMC.

The microstructure of HDMC is examined from SEM images. Figure 12a shows the matrix of the non-immersed HDMC with 7-day ambient curing. It can be seen that the struvite-K crystals in the matrix are mainly wedge-shaped and arranged closely. Figure 12b shows the bonding between fibers and the matrix of the non-immersed HDMC with 7-day ambient curing. It can be seen from the fibers that the cross-section of fibers does not change obviously, and there are some scratches, grooves, and hydration products on the surface of fibers, which indicates that the bonding between fibers and the matrix is relatively good, and the fibers may not be fractured but be pulled out from the matrix.

**Figure 13:**
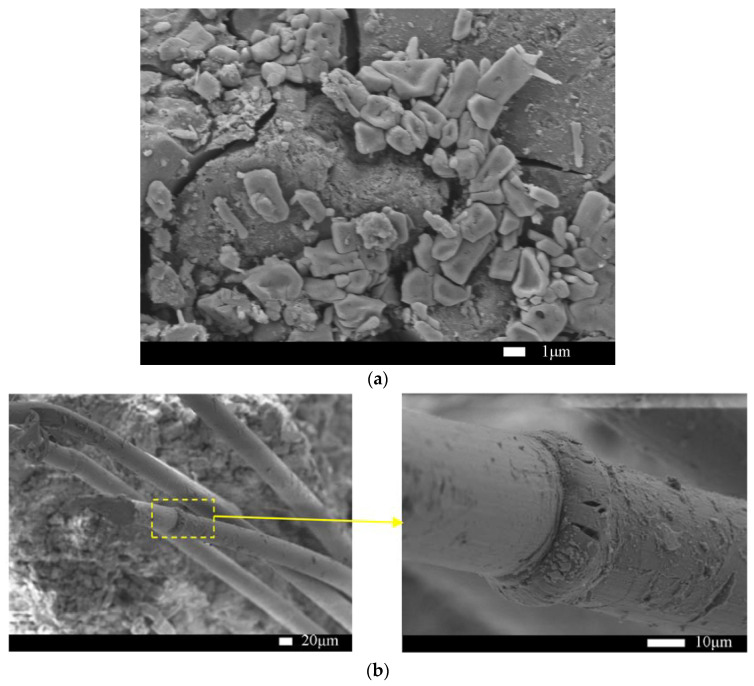
(**a**) The matrix and (**b**) its bonding with fibers for HDMC with 7-day water immersion.

Figure 13a shows the matrix of the HDMC with 7-day water immersion following 7-day ambient curing. It can be seen that there are many small substances attached to struvite-K crystals, which are believed to be unreacted MgO or MgO obtained from struvite-K decomposition. Therefore, the struvite-K generated by the hydration reaction is relatively less, and the gap between the crystals is relatively larger, which shows that the 7-day water immersion has a large adverse effect on the matrix, and the mechanical properties of the HDMC can be decreased. This is consistent with that the compressive strength (42.5 MPa) of the HDMC with 7-day water immersion is lower than that of the non-immersed HDMC by 5.1%. Figure 13b shows the bonding between fibers and the matrix of the HDMC with 7-day water immersion. It can be seen that the gap between the fibers and the matrix becomes larger after water immersion, and the bonding is not good enough. The surface of fibers is smoother than that of the fibers in the non-immersed HDMC, and there are not large amounts of hydration products attached. The possible reason is that the unreacted potassium dihydrogen phosphate dissolves after water immersion and makes the solution weakly acidic. Then the hydration reaction proceeds in reverse, which leads to the decomposition of the hydration product, struvite-K. This is consistent with that after 7 days of water immersion, the ultimate flexural strength of the HDMC decreases slightly.

## 4. Conclusions

In this study, magnesium phosphate cement (MPC), PVA fibers, and fly ash were combined to prepare high ductility magnesium phosphate cement-based composites (HDMC). The effects of ambient curing age, water immersion age, magnesium oxide/potassium dihydrogen phosphate (M/P) ratio, and water/binder (W/B) ratio on the water stability and mechanical properties of the HDMC were examined. Several important conclusions can be drawn.

For the HDMC with the same water immersion age, the 28-day ambient curing can lead to higher retention rates of strength, ductility, and toughness than 7-day ambient curing, indicating that the extension of ambient curing age can enhance the water stability of HDMC; however, a longer ambient curing age does not result in significant improvement in the mechanical properties of HDMC.

As the water immersion age increases from 0 to 56 days, the mechanical properties of the HDMC with 7-day ambient curing shows an obvious downward trend; however, the mechanical properties of the HDMC with 28-day ambient curing does not show an obvious decrease and even can be increased in many cases, especially when the water immersion age is 56 days. In terms of water stability, its change is consistent with that of the mechanical properties.

With the increase of the W/B ratio, the changes of strength index, ductility index, and toughness index are complicated. For non-immersed HDMC, the strength index, ductility index, and toughness index all can be satisfactory when the W/B ratio is 0.16. For immersed HDMC, the strength index is the best when the W/B ratio is 0.14, and the ductility index and toughness index are the best when the W/B ratio is 0.20; these three indexes are moderate when the W/B ratio is 0.16. The retention rates of strength, ductility, toughness seem to be the highest when the W/B ratio is 0.20, indicating the best water stability, followed by the W/B ratio of 0.16. Therefore, if all indexes and their corresponding retention rates are considered comprehensively, the W/B ratio of 0.16 seems to be the optimum value for HDMC.

With the increase of the M/P ratio, although the changes of the strength index, ductility index, and toughness index are not always consistent, it can be seen that for both non-immersed and immersed HDMC, the strength index, ductility index, and toughness index are satisfactory when the M/P ratio is 5. However, the retention rates of these indexes indicate that the HDMC with the M/P ratio of 5 shows moderate water stability, and the best water stability can be reached when the M/P ratio is 3.

The microstructure analysis indicates that for non-immersed HDMC, the struvite-K crystals in the matrix are mainly wedge-shaped and arranged closely; the bonding between fibers and the matrix is relatively good; and the fibers may not be fractured but be pulled out from the matrix. However, for immersed HDMC, the gap between the struvite-K crystals is relatively large, and the surface of fibers is smooth, with no large amounts of hydration products attached, which shows that the water immersion has a large adverse effect on the HDMC and thus reduce their mechanical properties.

## Figures and Tables

**Table 1:** Chemical composition of dead burned magnesia.

Chemical Composition	Mass Percent (%)
MgO	≧96
Fe_2_O_3_	≦0.18
SiO_2_	≦1.4
CaO	≦1.4
Al_2_O_3_	≦0.3

**Table 2:** Physical properties of M, P, and B.

Raw Materials	Particle Size (μm)	Specific Surface Area (m^2^/kg)	Appearance	Purity	Manufacturer
M	-	315.7	Light yellow powder	≥96%	Huanai [40]
Coarse P	425–600	-	White crystal	≥99%	Dingshengxin [41]
Fine P	180–315	-	White crystal	≥99%	Weitong [42]
B	-	-	White powder	≥99.5%	Banda [43]

Note: the missing content has not been tested and provided by the manufacturer.

**Table 3:** Technical indicators of quartz sand.

SiO_2_ Percent	Bulk Density	Mohs Hardness	Porosity	Specific Gravity
99.3%	1.8 g/cm^3^	7.5	43%	2.66 g/cm^3^

**Table 4:** Chemical composition of fly ash (FA).

Chemical Composition	Mass Percent (%)
SiO_2_	53.97
Al_2_O_3_	31.15
Fe_2_O_3_	4.16
CaO	4.01
MgO	1.01
Na_2_O	0.89

**Table 5:** Performance indicators of PVA fibers.

Diameter (μm)	Length (mm)	Tensile Strength (MPa)	Modulus of Elasticity (GPa)	Elongation at Break (%)	Density (g/cm^3^)
40	12	1560	41	6.5	1.3

**Table 6:** Experimental mix proportion.

W/B	M/P	S/B	Fly Ash Content	Borax Dosage	Fiber Volume Fraction
0.14	4	0.2	20%	6%	1.6%
0.16					
0.18					
0.20					
0.16	3	0.2	20%	6%	1.6%
	4				
	5				
	6				

## Data Availability

The data presented in this study are available on request from the corresponding author. The data are not publicly available due to some privacy.
